# Naloxone Ameliorates Spatial Memory Deficits and Hyperthermia Induced by a Neurotoxic Methamphetamine Regimen in Male Rats

**DOI:** 10.31661/gmj.v0i0.1182

**Published:** 2019-04-15

**Authors:** Solmaz Khalifeh, Mehdi Khodamoradi, Vahid Hajali, Hamed Ghazvini, Lelia Eliasy, Afshin Kheradmand, Vahid Farnia, Javad Akhtari, Kaveh Shahveisi, Hossein Ghalehnoei

**Affiliations:** ^1^Cognitive and Neuroscience Research Center (CNRC), Amiralmomenin Hospital, Tehran Medical Sciences, Islamic Azad University, Tehran, Iran; ^2^Substance Abuse Prevention Research Center, Kermanshah University of Medical Sciences, Kermanshah, Iran; ^3^Quchan Higher Health Education Center, Mashhad University of Medical Sciences, Mashhad, Iran; ^4^Psychiatry and Behavioral Sciences Research Center, Addiction Institute, Mazandaran University of Medical Sciences, Sari, Mazandaran, Iran; ^5^Molecular and Cell Biology Research Center, Faculty of Medicine, Mazandaran University of Medical Sciences, Sari, Iran; ^6^Department of Anatomical Sciences, Golestan University of Medical Sciences, Golestan, Iran; ^7^Department of Pharmacology and Toxicology, school of pharmacy, International campus, Iran University of medical sciences, Tehran, Iran; ^8^Immunogenetic Research Center, Mazandaran University of medical science, Sari, Iran; ^9^Sleep Disorders Research Center, Kermanshah University of Medical Sciences, Kermanshah, Iran

**Keywords:** Methamphetamine Hydrochloride, Naloxone Hydrochloride, Spatial Memories, Hyperthermia

## Abstract

**Background:**

Methamphetamine (METH) as a synthetic psychostimulant is being increasingly recognized as a worldwide problem, which may induce memory impairment. On the other hand, it is well established that naloxone, an opiate antagonist, has some beneficial effects on learning and memory. The present research aimed at evaluating naloxone effects on spatial learning and memory impairment triggered by a neurotoxic regimen of METH in male rats.

**Materials and Methods:**

The animals received the subcutaneous (sc) regimen of METH (4×6 mg/kg at 2-h intervals), intraperitoneal (ip) naloxone (4×1 mg/kg at 2-h intervals), or normal saline at four events. The Nal-METH group of rats received four naloxone injections (1 mg/ kg, ip) 30 min before each METH injection (6 mg/kg, sc) at 2-h intervals. Seven days later, they were evaluated for spatial learning and memory in the Morris Water Maze (MWM) task.

**Results:**

METH regimen induced hyperthermia, as well as a poor performance, in the acquisition and retention phases of the task, indicating spatial learning and memory impairment compared to the controls. Naloxone administration (1 mg/kg, ip) before each METH injection led to significant attenuations of both hyperthermia and METH adverse effects on the rat performance in the MWM task.

**Conclusion:**

The results revealed that pretreatment with the opiate antagonist naloxone could prevent METH adverse effects on body temperature and memory performance. It seems that the opioidergic system and hyperthermia may, at least partially, be involved in METH effects on spatial memory.

## Introduction

As a potent psychostimulant associated with extensive adverse effects on the nervous system, methamphetamine (METH) is being increasingly abused throughout the world, thus imposing a serious health concern on the world’s population [1, 2]. As increasingly mentioned in the literature, METH abusers are at a high risk of learning and memory impairment in the form of long-term cognitive dysfunction [3-6]. Accordingly, animal studies have shown that the chronic or toxic METH regimen can produce severe impairments in hippocampal-dependent tasks, particularly those of spatial memory[7]. It is also worthy of note that METH administration may induce hyperthermia, a condition which may lead to memory impairment [8, 9]. It is well documented that METH triggers dopamine release and there is a relationship between the opioidergic and dopaminergic systems in the nervous system. As previously reported, the opiate agonists and/or antagonists may modulate the function of the dopaminergic neurons [10]. The potent opiate antagonist naloxone has been shown to have analgesic effects via the opioid receptors [11]. For example, naloxone has been reported to modulate the response rate and threshold for rewarding the effects of psychostimulant amphetamine [10]. Naloxone may also attenuate METH-induced hyperthermia in mice in the same way as the effect of the dopamine receptor antagonist haloperidol [12]. Interestingly and importantly, a survey of the literature reveals that the opioidergic system may modulate the neural circuits involved in learning and memory, especially in the hippocampus [13-15]. Furthermore, considerable evidence suggests that naloxone can ameliorate cognitive function in both spatial and non-spatial memory tasks [16, 17]. Regarding the behavioral outcomes, it has been elucidated that naloxone enhances the performance of rats in the radial arm maze task, indicating improvement of the spatial working memory [13]. Upon this evidence, the current research aimed at figuring out if the opiate antagonist naloxone has any possible effects on spatial learning and memory dysfunction, as well as on hyperthermia caused by a neurotoxic regimen of METH in male rats.

## Materials and Methods

### Animals

Twenty-eight male Wistar rats weighing from 200 to 250 g were obtained from the maintained colony at Pasteur Institute (Tehran, Iran). Caging of the rats in groups of 4 animals at a temperature of 23±1 ºC and a light/dark cycle of 12:12 h was followed with daylight beginning at 07:00 am. The animals were provided with ad libitum food and water. All the protocols and ethical issues were conducted according to the ethics committee of Tehran University of Medical Sciences (970327).

### Experimental Protocols

The rats were randomly divided into 4 groups (n=7 in each group), while receiving one of following 1-day treatments: 1) The control group received 4 subcutaneous (sc) normal saline injections at 2-h intervals; 2) the METH group was administered with the subcutaneous injection of METH (Catalog ID M8750; Sigma-Aldrich Co., St. Louis, MO) dissolved in normal saline at 4 events (4×6 mg/kg at 2-h intervals) [7]; 3) the Nal group received the intraperitoneal (ip) injection of naloxone (Sigma-Aldrich Co., St. Louis, MO) dissolved in normal saline at 4 events (4×1 mg/kg at 2-h intervals) [13]; and 4) the Nal-METH group were injected with METH (6 mg/kg, sc) and naloxone (1 mg/kg, ip) at 2-h intervals. In the last group, the animals were administered with naloxone 30 min prior to each METH injection [12, 18]. The animals were evaluated in the Morris Water Maze (MWM) task for spatial learning and memory one week later.

### Body Temperature (BT) Measurement

Immediately after each administration, a lubricated and flexible rectal probe (for 40 s) connected to a digital thermometer (LE0331 Panlab SL, Barcelona, Spain) was inserted into the rectum to measure of the core BT [19].

### Morris Water Maze (MWM)

The MWM task was conducted according to the previous research [20-22]. A circular metal tank with the diameter and wall height of 160 and 80 cm, respectively, was filled with water to a depth of 40 cm, while the water temperature was constantly maintained at 22±1 ºC. After geographically dividing the tank into four equal quadrants, a platform with a diameter of 10 cm was placed ~1.5 cm beneath the water surface in the middle of one of them to serve as the target quadrant. A video tracking system (Noldus Ethovision XT, version 5, USA) was employed to record the swimming paths of the rats, while the camera was placed directly above the maze. The animals learned to discover the hidden platform using the squares, triangles, and circles acting as the visual (spatial) extra-maze stimuli, which were attached onto the experimental room walls. The animals tried their tasks with 3 training blocks and 30-min intervals of rest period during the acquisition phase. Each block was attempted with four successive trials and intervals of 60 s. In each trial, they were permitted to swim for 60 s after being gently dropped into the water at the center of the randomly specified target quadrant. They were allowed to stay on the platform for 20-30 s after discovering it. In case they did not find it, they were gently directed towards the platform to rest during the specified time. Then, they were put back into the cages under a heating lamp for 30-35s till the next trial. The spatial learning was analyzed by determining the spent time (mean escape latency) for discovering the hidden platform with regard to the traveled distance (mean path length). The spatial short-term memory was evaluated after completing the learning phase and conducting the 2-h retention phase of probe trials by removing the hidden platform, dropping the animals into the water from the quadrant located opposite the target quadrant, and allowing them to swim for 60 s. The indices of mean escape latency, mean path length, and number of crossing the center of the target quadrant were measured to evaluate the spatial short-term memory. Finally, a visible platform elevated ~2 cm over the water surface was employed to measure the spent time for finding it within 60 s so as to determine any possible effects of METH and/or Nal-METH administrations on the visual, sensory (perceptual), and motor performance of the rats.

### Data Analysis

All the data were analysed via SPSS 18 software (SPSS Inc., Chicago IL, USA). The core BT data were analyzed based on the repeated measures ANOVA. The two-way analysis of variance (ANOVA) was utilized to analyze the data (mean escape latency and mean path length) of the acquisition trials after the hidden platform was discovered by the experimental groups across the training blocks. The one-way ANOVA was applied to assess the data of the retention (mean escape latency, mean path length, and number of crossing the target quadrant), visible platform (escape latency towards the platform), and swimming speed tests. All the post-hoc tests were carried out based on the Tukey’s test for multiple comparisons. The data were expressed as means±Standard Error of the Mean (SEM).

P<0.05 was considered statistically significant.

## Results

### BT Measurement

**Figure 1:**
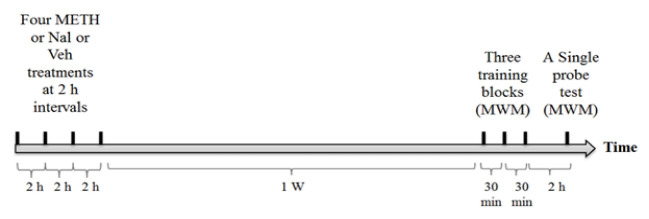
The timeline and protocol of the study. The animals received four administrations of METH or naloxone or vehicle at 2-h intervals. Naloxone or Veh was injected 30 min prior to each METH administration. Their spatial learning and memory were assessed one week later using the Morris water maze task. METH: methamphetamine; Nal: naloxone; Veh: vehicle; MWM: Morris water maze; h: hour; W: week.

Figure-1 shows that METH administration to the METH group at the ambient temperature of 21±1 ºC induces hyperthermia as compared to the control group (P<0.001), especially after four injections. On the other hand, the animals of the Nal group have not significant difference from those of the control group. After measuring BT in the Nal-METH group 4 times, METH administration was found to have significantly reduced hyperthermic responses in the METH compared to the control group (P<0.05).

### Spatial Learning

**Figure 2:**
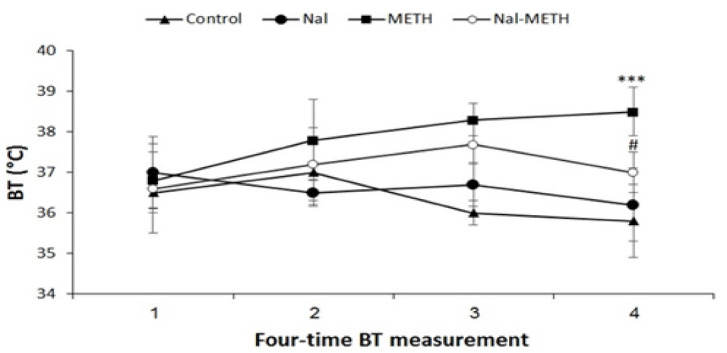
Evolution of the body temperature after each (four) treatment(s) in the different groups. A neurotoxic METH regimen (four sc injections of 6 mg/kg METH at 2-h intervals) in the METH group significantly enhanced body temperature compared with the saline-treated animals (control group). Pre-treatment with naloxone (1 mg/kg, i.p.) 30 min before each METH injection (in the Nal-METH group), but not naloxone alone (in the Nal group), significantly reduced the body temperature in comparison with the METH group. Data are expressed as mean ± S.E.M. ***P<0.001 vs. control group; # P<0.05 vs. METH group.

The acquisitions of the experimental rats (learning to discover the hidden platform) revealed significant reductions of the escape latencies and path lengths across the training blocks (Figure-2). The analysis of variance was indicative of significant increases in the spent times and traveled distances in the first 3 blocks (P<0.001) in the METH compared to the control group. However, no significant differences in the two mentioned indices were found in the Nal compared to the control group. Moreover, the results displayed significantly reduced escape latency and path length for the Nal-METH group to find the hidden platform when naloxone was administered to them prior to each METH injection in comparison with the METH group. The analysis further demonstrated significant reductions of the mean distance traveled by the animals of the Nal-METH group and their mean time spent in block 1 (P<0.05 and P<0.01 for the traveled distance and escape latency, respectively), block 2 (P<0.01), and block 3 (P<0.05) as compared to those of the METH group (Figure-2).

### Spatial Short-term Memory

**Figure 3:**
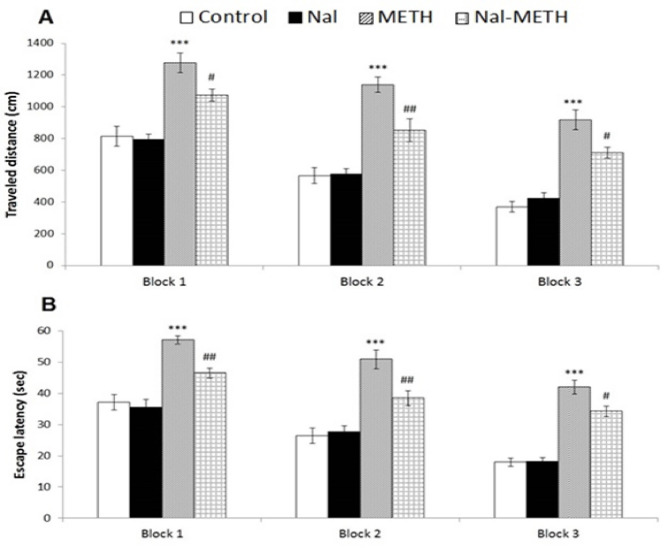
Performance of the animals in the acquisition phase of the MWM; path length (A) and escape latency (B) to discover the hidden platform. A neurotoxic regimen of METH (four sc injections of 6 mg/kg METH at 2-h intervals) in the METH group significantly increased both path length and escape latency toward the hidden platform across blocks of training in comparison with the control group. Naloxone pre-treatment (1 mg/kg, i.p.) before each METH administration (in the Nal-METH group), but not naloxone alone (in the Nal group), significantly decreased both path length and escape latency compared with the METH group. Data are shown as mean ± S.E.M. ***P<0.001 vs. control group; # P< 0.05, and ## P< 0.01 vs. METH group.

**Figure 4:**
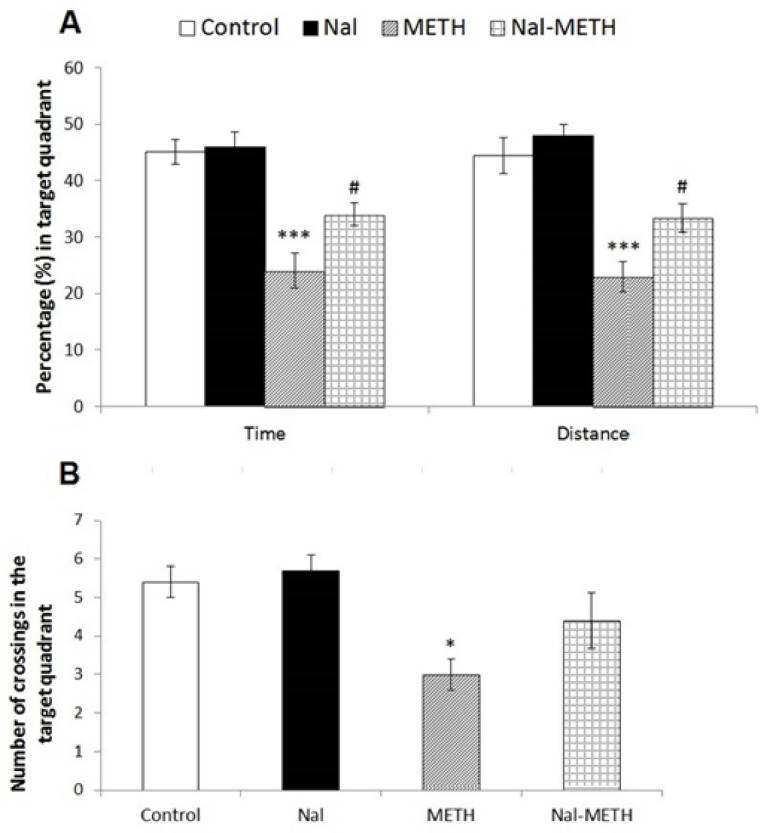
Performance of the animals in the retention phase of the MWM; the time spent and traveled distance (A) and also the number of crossings (B) in the target quadrant. METH regimen (four sc injections of 6 mg/kg METH at 2-h intervals) in the METH group significantly decreased the time spent, traveled distance, and the number of crossings in the target quadrant than those of the control group. Naloxone pre-treatment 30 min before each METH injection (in the Nal-METH group), but not naloxone alone (in the Nal group), significantly enhanced the time spent and traveled distance, but not the number of crossings, in the target quadrant compared with the METH group. Data has been shown as mean±S.E.M. *P<0.05 and ***P<0.001 vs. control group; # P< 0.05 vs. METH group.

Short-term memory performance was assessed by analyzing the escape latency, path length, and number of crossing the target quadrant in the probe test conducted 2h after the training phase. Figure-3 exhibits no significant differences between the rat performance in the Nal group (P>0.05) and the control group through the one-way ANOVA. On the other hand, the results were indicative of the poor performance of the METH group in memory retention after receiving a neurotoxic regimen of METH since they showed significantly less spent time (P<0.001) and traveled distance (P<0.001, Figure-3A), as well as fewer numbers of crossing the target quadrant (P<0.05, Figure-3B) than the control group. The analysis also demonstrated that treatment with naloxone before each METH administration significantly reduced impairments in spatial short-term memory retention. Thus, the animals of the Nal-METH group significantly spent more times (P<0.05) and traveled farther distances (P<0.05) across the target quadrant compared to the METH group (Figure-4A). Also, the METH group displayed significantly fewer numbers of crossing the target quadrant (P<0.05) as compared to the control group though these results were not significantly different from those of the Nal-METH (Figure-4B).

### Swimming Speed and Latency of Visible Platform Discovery

As indicated by the analysis, no significant differences were found between the groups in terms of swimming speed and escape latency to discover the visible platform (P>0.05, Table-1). Therefore, the findings indicated that METH and naloxone treatments did not interfere with the swimming performance of the animals.

## Discussion

In the present study, the possible effects of the potent opiate antagonist naloxone on the hyperthermia and subsequent cognitive dysfunctions of male rats were examined in the MWM task following a neurotoxic METH regimen. Our findings demonstrated that pretreatment with the opiate antagonist naloxone may improve METH-induced hyperthermia and spatial short-term learning and memory deficits. These results provided a novel insight into the opioidergic system involvement in the negative effects of the psychostimulant METH on cognition. Animal’s studies are more important for cognitive and behavioral interventions [23-28]. The findings of this research are in line with those of the previous animal’s studies regarding METH adverse effects on memory [4, 29, 30]. It was shown that a neurotoxic regimen of METH not only induced damage at cellular levels but also impaired the performance or the rats in the novel object-recognition task and multiple T-water-maze test of path integration [29]. It was reported that both the neurotoxic METH regimen and METH withdrawal after 14 days could result in memory deficit and impairment of hippocampal synaptic plasticity (long-term potentiation [LTP]) [3, 7]. It is worth noting that hippocampal LTP has been introduced as a model for memory [31]. It was also demonstrated that damage to the dopaminergic and/or serotoninergic neurons in the striatum, hippocampus, and perirhinal cortex, as well as neuronal degeneration in the somatosensory cortex, may, at least partially, be responsible for object recognition memory deficit following administration of the METH neurotoxic regimen [32]. METH might cause dopaminergic neurotoxicity and memory impairment by inducing extreme hyperthermia as well [8, 9]. Moreover, it was reported that a neurotoxic regimen of METH-induced object-recognition memory deficits in young male rats, but it did not affect spatial memory in the MWM task [32]. Conversely, Ghazvini *et al.* [7] reported METH adverse effects on spatial memory. Their results revealed spatial learning and memory deficits caused by a neurotoxic regimen of METH in the MWM task. Our experiments led to hyperthermia and spatial learning and memory impairment induced by the neurotoxic regimen of METH in male rats, the results of which are in agreement with those of some previous studies. Thus, the assumption of complicated METH effects on spatial learning and memory seems to be reasonable though they may be partially attributable to hyperthermia and dopaminergic neurotoxicity. Our results further indicated that pretreatment with the opiate antagonist naloxone improved spatial memory deficits following METH administration. These findings are compatible with those of the previous studies demonstrating the beneficial effects of opiate antagonists on cognition and neuronal function [13, 33]. It is well established that the opioidergic system is involved in the hippocampal-dependent tasks in such a way that opiate antagonists and agonists improve and impair spatial learning, respectively [16, 34]. Decker *et al*. [34] showed that pre-training naloxone administration enhanced spatial learning and memory, while its post-training administration did not affect the performance of the animals in the MWM task. It was elucidated that the opiate antagonist naloxone ameliorated memory in both active and passive avoidance tasks [33]. In the present research, the naloxone-treated animals displayed performance improvement following spatial learning and memory deficits induced by METH. This result is congruent with those of the studies mentioned above. Another interesting finding in this study was that naloxone decreased METH-induced hyperthermia. The morphine antagonist naloxone was shown to prevent hyperthermia induced by the administration of METH plus morphine in mice [12]. It was also stated that morphine induces changes in BT and naloxone may suppress temperature changes due to stress [35]. It should be noted that morphine had a dual action leaving hyperthermic and hypothermic effects on the non-stressed and stressed animals depending on their stress levels, respectively [36]. It is also interesting to mention that naloxone may decrease hyperthermia via acting on the opioid receptors as it has been reported to reduce hyperthermia induced by conditional stimulus in male rats [37]. Thus, according to our findings, as well as those of the previous studies, it is possible that naloxone alleviates the hyperthermic effects of METH, at least partially, via lowering stress levels and acting on the opiate receptors. The precise mechanisms of naloxone effects on memory are unclear. From this point of view, it has been stated that naloxone-induced improvement of performance in the MWM task is accompanied by an enhanced LTP in the hippocampal slices [38]. Therefore, it can be suggested that naloxone can, in part, improve cognitive function through hippocampal synaptic plasticity enhancement. It has also been reported that naloxone antagonizes the inhibitory actions of dynorphins on the glutamatergic system and thereby enhances hippocampal synaptic plasticity [39]. Moreover, naloxone decreases microglial activation and reduces the production of microglial O2, thus exerting neuroprotective effects [40]. Interestingly, Galea *et al*.[16] reported the sex-dependent effects of naloxone as it had increased spatial acquisition in female meadow voles, but it had not affected the performance of male meadow voles in the MWM task. Therefore, it may be suggested that stress and the levels of gonadal steroids can augment the sex-dependent effects of naloxone on cognition. It is possible that the mechanisms mentioned above have been, at least partially, involved in naloxone effects on METH-induced memory deficits in the present research. Furthermore, the probable sex-dependent effects of naloxone on METH-induced memory impairment can be of some interest for future investigations.

Our findings importantly demonstrated that the visual, sensory, and motor performances of the studied rats in the MWM task were not influenced by the applied treatments as no differences between the groups were observed after performing the visible test. The results suggested that naloxone effects on METH-induced hyperthermia and cognitive dysfunction could not be attributable to the probable methodological problems.

## Conclusion

The present study demonstrated that hyperthermia and the opioidergic system may be involved in the adverse effects of METH administration on spatial learning and memory impairment. Thus, it might be suggested that the pharmacological treatments, which can target the opiate receptors and reduce hyperthermia, are capable of improving the negative effects of the psychostimulant METH. Additionally, our results indicated that hyperthermia might play a significant role in METH-induced memory deficit; however, the precise mechanisms, through which hyperthermia induces memory deficit remain to be clarified.

## Acknowledgment

The authors would like to thank Shafagh Shariati for critical proofreading ofthe manuscript.

## Conflict of Interest

The authors declare that they have no conflicts of interest.

**Table 1:** Swimming Speed and Latency to Find the Visible Platform

**Groups**	**Swimming speed (cm/s)**	**Escape latency (s)**
**Control** **Nal** **METH** **Nal-METH**	21.43 ± 1.63 22.29 ± 3.01 20.95 ± 2.61 21.70 ± 2.52	19.36 ± 2.34 20.12 ± 1.33 18.36 ± 3.12 17.14 ± 2.05

ANOVA test revealed no any significant difference between the groups. Data are expressed as mean ± S.E.M.
